# Optimizing weathering steel tie rod production for sustainable greenhouse structures

**DOI:** 10.1038/s41598-026-45791-9

**Published:** 2026-04-27

**Authors:** Maha El-Meligy, Taher El-Bitar, Almosilhy Mohammed

**Affiliations:** Plastic Deformation Department, Central Metallurgical R&D Institute (CMRDI), Cairo, 11422 Egypt

**Keywords:** Sustainable processing technology, Structure steel, Tie rod, Weathering steel, Corrosion index, Greenhouse structure, Engineering, Materials science

## Abstract

The current paper is based on a case study conducted at an industrial facility in Egypt, which is responsible for processing greenhouse structures. Steel tie rods play a vital role, as they prevent the truss frames from spreading outwards. Irrigation and plant transpiration generate water vapor that condenses on truss parts, accelerating corrosion and degrading the mechanical performance of the truss system. To enhance durability under these conditions, the study investigates the use of weathering steel, a copper-alloyed structural steel known for its long-term corrosion resistance. Furthermore, the study investigates the flanging process used to create tie rod ends, where uneven flange thickness and non-homogeneous deformation have been observed due to deformation in the unstable region of the hot-working map. The process was conducted using a 14-ton press at 700 °C with a strain rate of 0.4 s⁻¹. It was found that a steel composition containing 0.37% Cu with phosphorus provides optimal resistance to weathering and humid conditions. The processing map revealed stable deformation regions between 1000 and 800 °C, with an optimal hot-working window at 850 °C and a strain rate of 0.03 s⁻¹. The integration of weathering-steel alloying with optimized processing parameters significantly improves durability, stability, and sustainability.

**Supplementary Information:**

The online version contains supplementary material available at 10.1038/s41598-026-45791-9.

## Introduction

Sustainable water management is a major global challenge, as freshwater resources are becoming increasingly scarce in many regions. This scarcity has significant political, social, and economic implications^1^. Agriculture is the largest consumer of freshwater worldwide, accounting for an average of 70% of all withdrawals. In developing countries, this proportion can reach up to 95%, placing immense pressure on available water resources. Egypt, as a developing country, faces a particular challenge in providing sufficient irrigation for agricultural production.

Modern cultivation systems, such as greenhouse agriculture, help reduce water loss through transpiration. In contrast to traditional irrigation methods, plants can lose up to 98% of the water they absorb, leading to major inefficiencies in water use^1^.

**Fig. 1:**
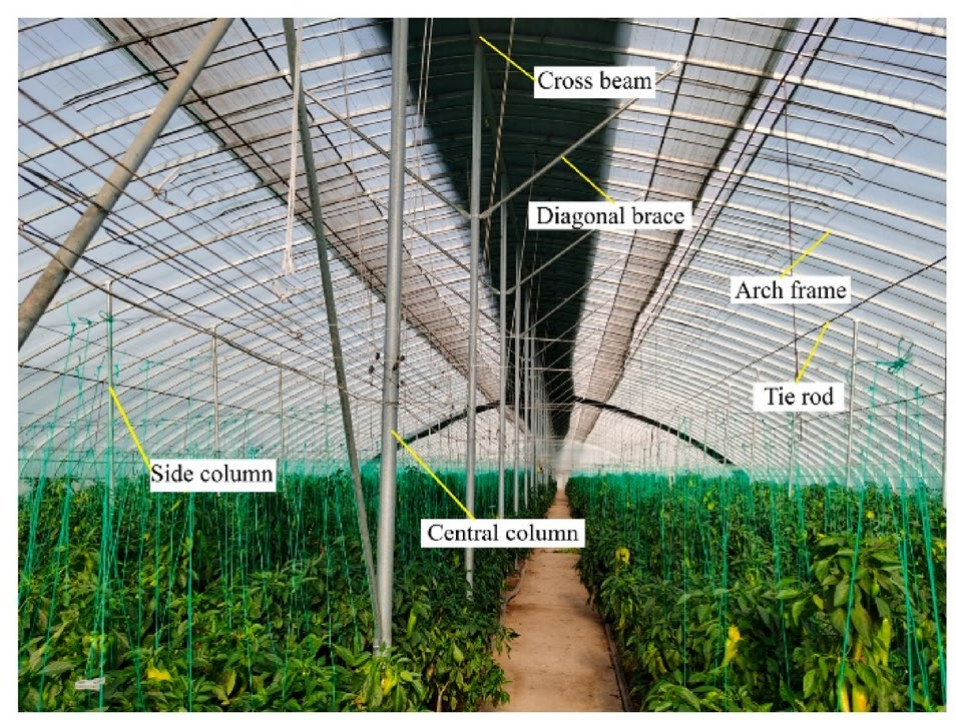
Greenhouse truss system and tie rod location^2^.

**Fig. 2:**
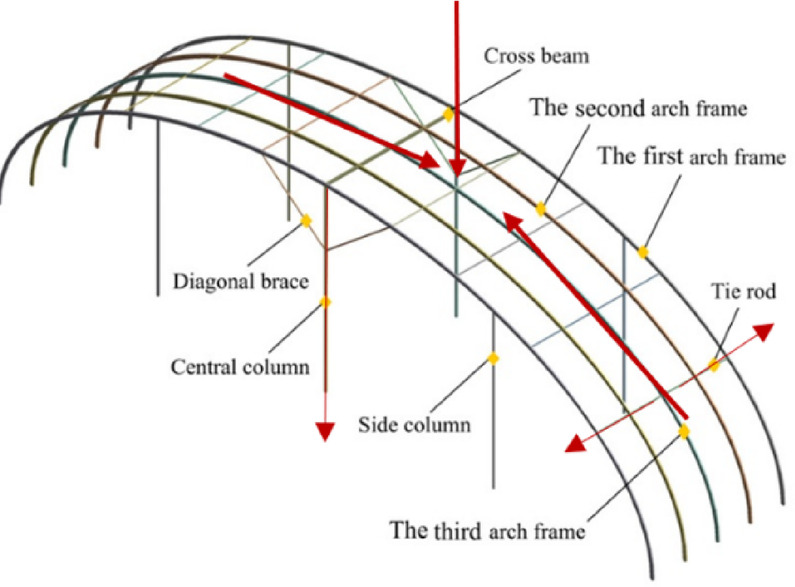
Schematic diagram of a truss showing applied loads on each member^2^.

A greenhouse is a structure assembled from multiple components, each serving a specific role within the overall system. Among these components, the steel tie rod is a critical element in structural roof trusses, preventing the bottom sections from spreading outward. Figure 1 greenhouse truss system and tie rod location. Tie rods may include flanges to facilitate connections with other structural elements, providing a wider and more secure attachment point. The flange at the end of the tie rod allows for bolted connections to the truss components. The greenhouse framework, which shows arch frames, columns, diagonal braces, cross beams, and the tie rod. Roof loads produce compressive stresses in the arches, generating outward thrust at their bases. The tie rod resists this thrust by carrying tensile forces along its length, connecting the lower arch joints and maintaining structural stability under service loads. Figure 2 Schematic diagram of a truss showing applied loads on each member.

Low-carbon steel is widely used in greenhouse structures due to its excellent workability and ductility, making it suitable for bending and shaping in construction applications^3^. Greenhouse irrigation and plant transpiration generate high humidity, which condenses on cooler metal surfaces, creating ideal conditions for rust formation. Rapid temperature fluctuations further accelerate condensation.

Weathering steel, a low-alloy steel, provides enhanced corrosion resistance through the formation of a protective patina. Alloying elements such as copper (Cu) promote this layer, reducing maintenance requirements^4^. For example, Corten steel forms a patina that protects against severe corrosion, even in high-salinity environments^5^. Patina formation depends on environmental factors like humidity and exposure cycles, and develops faster in cold-rolled materials compared to hot-rolled ones.

Steel microstructure also affects corrosion behavior. Lamellar Fe₃C in pearlite accelerates ferrite dissolution and Fe²⁺ accumulation, while Fe₃C in martensite promotes localized corrosion by disrupting protective layers. Among common microstructures, bainite ferrite (BF) offers the highest corrosion resistance, followed by martensite ferrite (MF), with pearlite ferrite (PF) being the least resistant^6^.

Grain refinement induces microstructural changes—such as alterations in grain boundaries, dislocation density, texture, and residual stress—that influence corrosion behavior. An increase in grain boundary and dislocation density, resulting from reduced grain size, can enhance the formation of a protective passive film in passive systems or provide additional active dissolution sites in active systems, which may reduce corrosion resistance^7^.

The initial susceptibility to corrosion is often affected by grain size. Grain boundaries, which may contain impurities and exhibit lower activation energy for chemical reactions, serve as preferential sites for corrosion initiation. However, once a dense rust layer forms, the corrosion rate becomes more dependent on the properties of the rust itself, including its thickness and density, while the influence of the steel’s microstructure diminishes^8^.

Numerous computational models have been developed to predict the corrosion resistance of weathering steels. One such model, standardized in ASTM G101-16, defines a corrosion index (I) as a function of chemical composition. For effective corrosion resistance, the corrosion index should be equal to or greater than 6^9^;

1 $$\begin{aligned} I{\text{ }} & = 26.01\left( {\% Cu} \right) + 3.88\left( {\% Ni} \right) + 1.2\left( {\% Cr} \right) + 1.49\left( {\% Si} \right) + 17.28\left( {\% P} \right)~ \\ & - 7.29\left( {\% Cu} \right)\left( {\% Ni} \right) - 9.1\left( {\% Ni} \right)\left( {\% P} \right) - {\text{ }}33.39{\text{ }}\left( {\% Cu} \right)2 \\ \end{aligned}$$

Structural steel is a type of steel specifically designed for construction applications. It is available in a variety of shapes, each with distinct properties suited to specific structural requirements.

Forging is one of the oldest and most widely used metalworking processes. It involves shaping metal through compressive forces applied by hammers, presses, or dies. Process parameters play a critical role in ensuring components of ideal quality while maximizing mechanical properties.

Deforming steel near its Ac3 temperature (around 800 °C) produces a microstructure consisting of fine ferrite grains with some pearlite. Deformation above Ac3 (e.g., at 850 °C) results in a more uniform distribution of fine, equiaxed ferrite grains and finely dispersed pearlite. This indicates that grain refinement is most effective when deformation occurs close to the Ac3 transformation temperature^10,11^.

The deformation process not only shapes the raw material but also directly influences mechanical properties due to its impact on microstructure^12^. Optimizing the processing and properties of carbon structural steels requires a thorough understanding of the interactions between deformation temperature, holding time, and strain rate, and their influence on the dominant deformation mechanisms^13^.

Flow curves at high strain rates are essential for hot rolling of large plates, strips, and wire rods, as well as for forging and extrusion processes. During high-temperature deformation, two primary softening mechanisms occur: Dynamic Recovery (DRV) and Dynamic Recrystallization (DRX). DRX is a more complete recrystallization process that results in significant grain refinement, while DRV only partially rearranges the material’s internal structure^14^.

In DRX, deformed grains with high dislocation density are replaced by new, finer grains with lower dislocation density, enhancing both strength and ductility. The relationship between strain rate and DRX is critical for processing: higher strain rates alter the onset and progression of DRX. For example, in 0.2% carbon steel, strain rates above 10 s⁻¹ at high temperatures can delay the onset of DRX^15^.

a mixture of recrystallized grains and recovered, unrecrystallized sub-grains, resulting in moderate softening and slightly higher peak flow stress compared to very low strain rates, where recovery dominates^16,17^.

The processing of steel tie rods involves multiple steps, including hot pressing, material selection, heat treatment, and quality control. Each step must be carefully managed to ensure that the final product meets operational and safety requirements. This research aims to analyze these processing steps and identify methods for controlling the dimensions and mechanical properties of the finished tie rods.

### Problem definition

**Fig. 3:**
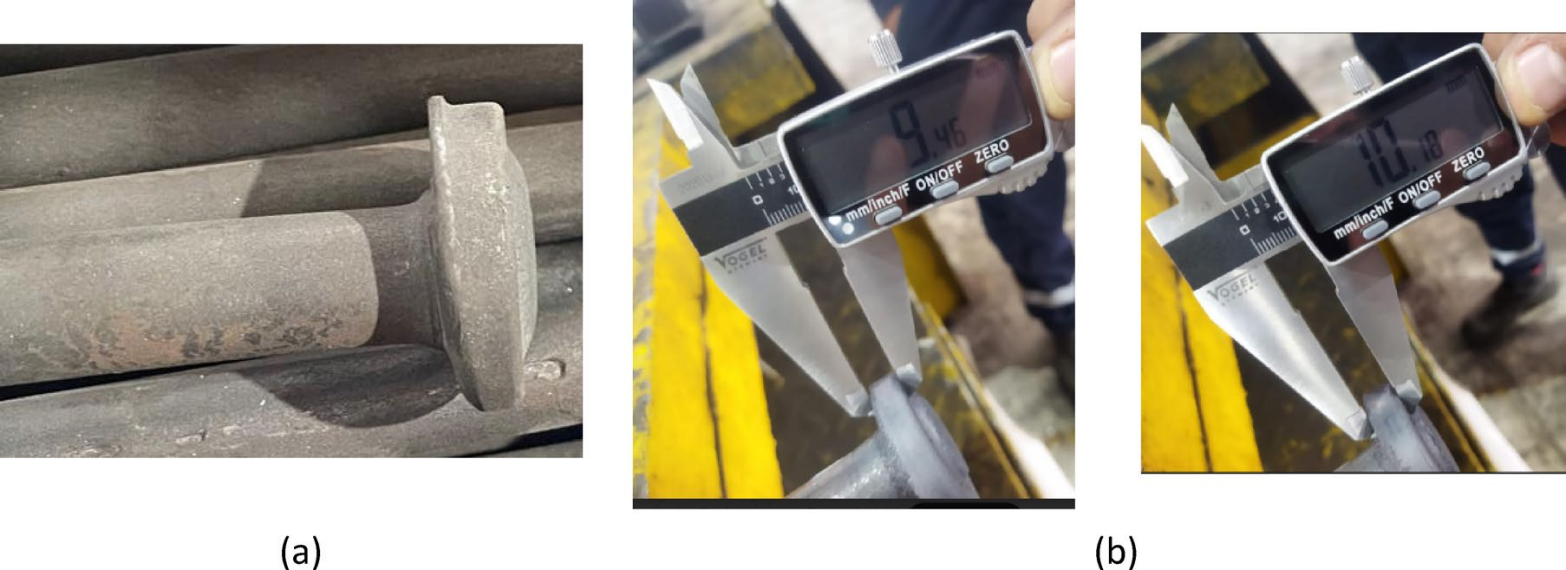
(**a**) Photo of the flange of the tie rod with uneven thickness. (**b**) Flange thickness variation with t_min_=9.46, t_max_= 10.18.

A quality issue was observed during the manufacturing of a tie rod flange at a greenhouse truss factory. The pressing process produced a flange with uneven thickness, which could compromise the structural stability of the roof truss. Figure 3a shows a photograph of the tie rod flange exhibiting this uneven thickness. Figure 3b illustrates the flange thickness variation, indicating the lowest(t_min_) and highest (t_max_) measured values.

The uneven flange thickness of the tie rods prompted an investigation into potential causes, including material properties and pressing process parameters. Achieving a successful steel pressing operation requires careful consideration of the material, tool design, and processing parameters to produce components with the desired shape, quality, and cost-effectiveness.

### Steel tie rod flange manufacturing process

**Fig. 4:**
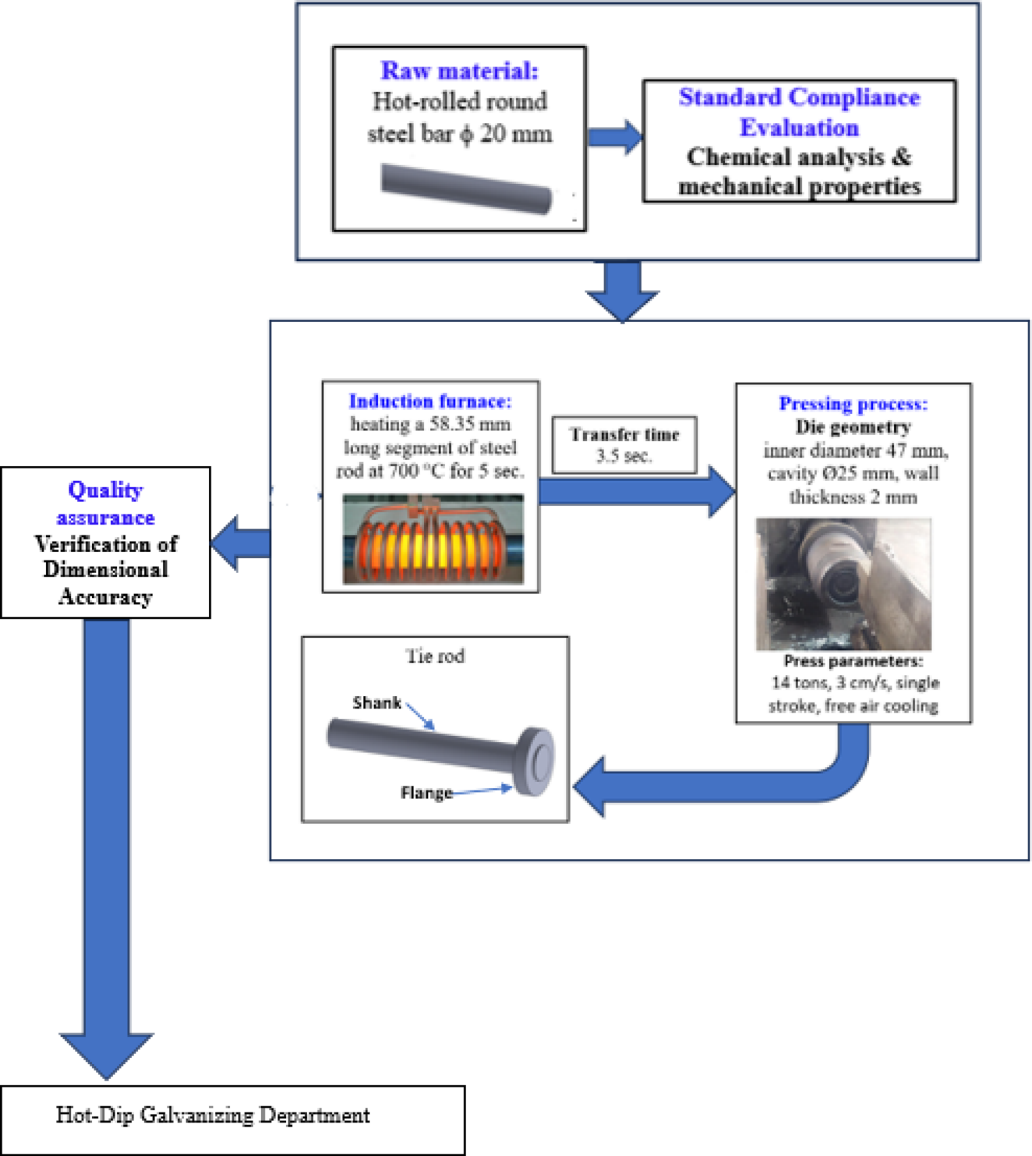
Schematic representation of the tie rod flange processing flow.

Figure 4 illustrates the steps involved in flange processing of the tie rod. The manufacturing process begins by heating one end of 20 mm diameter structural hot-rolled steel bars in an induction furnace at 700 °C for 5 s. The remaining length of the bar was not subjected to thermal exposure. The heated rods are then transferred to a pressing operation, followed by air cooling.

The press employs a die with a 47 mm inner diameter and a cavity designed with a 25 mm diameter and 2 mm wall thickness to form the final flange shape. A pressing force of 14 tons is applied at a speed of 3 cm/s.

**Fig. 5:**
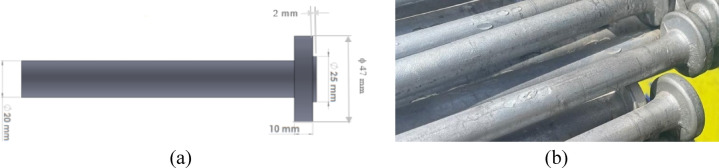
(**a**) Schematic engineering drawing for tie rod with flange. (**b**) A photo of a tie rod with flange end.

Figure 5a shows an engineering drawing of a tie rod with a ϕ20 mm spindle and a ϕ47 mm flange, providing detailed technical dimensions. Figure 5b presents a photograph of an actual tie rod with the flange end.

### Initial inspection

Die inspections confirmed that the components maintained geometric symmetry, with no evidence of misalignment or asymmetric wear that could account for the observed flange irregularities. While temperature gradients may develop during forging, the non-uniformity does not align with the primary thermal contact regions.

### Chemical analysis and material properties

Chemical analysis of the steel samples was performed using a Foundry Master Pro optical emission spectrometer (OES). Sample surfaces were prepared by cutting and grinding to ensure oxide-free surfaces. For each sample, three sparks were taken at different locations to account for local variation. The measurements were calibrated using certified reference materials. Supplementary Materials S1 illustrates Sample Preparation, Calibration, and Chemical Analysis Using Three Spark Tests. The reported chemical composition values in Table 1 represent the mean ± standard deviation of the three spark readings and are compared with the standard values specified in EN 10,025 (S355J2).

**Table 1:** Chemical composition values of the tube sample compared with the standard value of EN10025: S355J2.

Element, wt%	C	Si	Mn	*P*	S	Cr	Cu
Mean ± standard deviation	0.189 ± 0.006	0.189 ± 0.006	0.189 ± 0.006	0.189 ± 0.006	0.189 ± 0.006	0.189 ± 0.006	0.189 ± 0.006
EN10025: S355J2	Max 0.22	Max 0.55	Max 1.6	Max 0.035	Max 0.035	–	Max 0.55

**Fig. 6:**
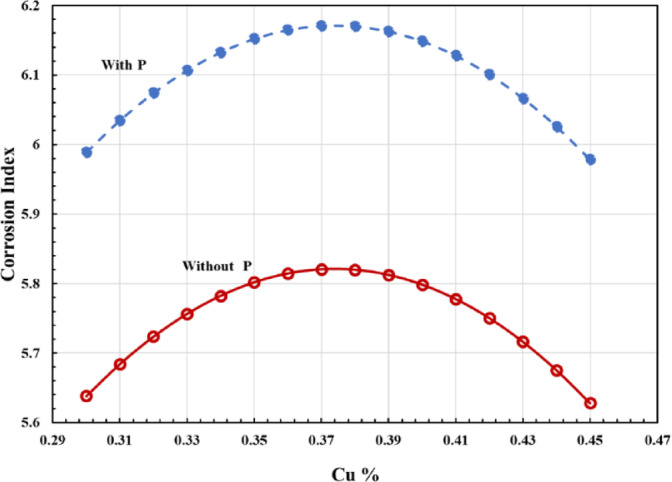
Impact of phosphorus (P) on the corrosion index of various alloys, as a function of copper content (Cu %).

The chemical composition of the rod sample coincides with a low-alloy structural steel grade EN 10,025: S355J2 (Material No. 1.0577). While copper (Cu) slightly reduces cold-workability and ductility, it enhances corrosion resistance by forming a dense protective patina, eliminating the need for painting. The corrosion index (I), calculated using Eq. (1), shows a clear correlation between Cu content and corrosion resistance, with enhanced performance observed when phosphorus (P) is present Fig. 6.

The corrosion index (I) of steel rises with copper content up to ~ 0.37 wt%, then declines, while phosphorus consistently enhances the index. Low Cu levels (< 0.37 wt%) promote formation of a thin, uniform protective oxide layer, whereas higher Cu levels (> 0.37 wt%) produce a thicker, rough CuO layer that reduces corrosion resistance^18–20^. Small variations in Cu (0.30–0.46 wt%) and P during production significantly affect the corrosion index, highlighting the need for strict compositional control to ensure predictable corrosion performance.

The study uses a corrosion index based solely on the steel’s chemical composition, which serves as an acceptance limit within the specification, providing only a preliminary indication of susceptibility rather than a full prediction of real-world corrosion rates. The material’s corrosion resistance relies on a dual protection mechanism. Galvanization offers primary protection, providing a continuous zinc layer over the steel surface. In addition, the steel’s copper–phosphorus content contributes localized corrosion resistance by forming a thin, adherent patina in areas where the coating is compromised, thereby slowing further degradation and enhancing the overall durability of the component.

Steel tie rod samples were subjected to tensile testing at room temperature (23 °C) in accordance with ASTM A53/A53M-99b. The tests were conducted using a crosshead speed of 10 mm/min. Test specimens were prepared in accordance with DIN 50,125 – A14 × 90. Specimen Geometry and Mounting in the Universal Testing Machine (UTM) Grips, as shown in Supplementary S2.

**Fig. 7:**
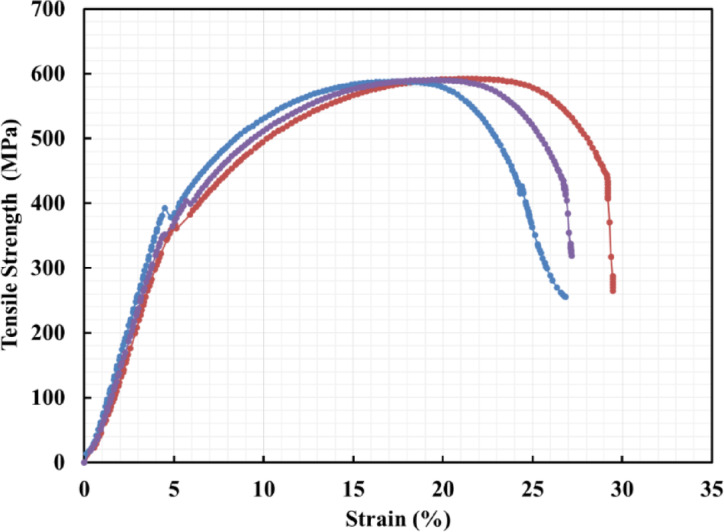
Stress-strain curve of steel rod samples under tensile test.

Figure 7 shows the tensile strength versus strain curves for the tested material samples.

Table 2 summarizes both individual and mean values obtained from these tests. The measured mechanical properties are consistent with the standard values specified for EN 10,025: S355J2 steel.

**Table 2:** Stress-strain curve of steel rod samples under tensile test.

Sample #	Yield stress (0.1%), *N*/mm^2^	Ultimate strength, *N*/mm^2^	Elongation, %
1	372.8	592.76	29.5
2	395.8	590.34	26.84
3	403.86	590.38	27.17
(Mean value) ± standard deviation	390.82 ± 16.12	591.16 ± 1.38	27.84 ± 1.45
Stand. values of EN10025: S355J2	355–550	490–630	Min 22%

### Hardness mapping

Hardness testing was performed using a NEMESIS 9104 hardness tester. The Vickers (HV10) method was employed, with a dwell time of 13 s for each measurement. Specimens were prepared according to standard metallographic procedures to ensure a smooth, flat surface for accurate indentation. Ten indentations were made to ensure reproducibility, and the results ranged from 166.37 HV10 to 175.17 HV10, with a mean hardness of 171.40 HV10 and a standard deviation of 2.86. This is confirmed with tensile test results. The observed variation is minimal, indicating good uniformity in the sample material. The full dataset, including individual measurements and statistical analysis, is summarized in Table 3. Supplementary S3 represents the hardness testing machine and the Test report.

**Table 3:** Vickers (HV10) hardness measurements of the sample.

#	1	2	3	4	5	6	7	8	9	10	Mean	SD
HV10	166.37	173.39	173.52	173.58	171.82	172.86	169.95	169.42	175.17	167.90	171.40	2.86

### Microstructure investigations

**Fig. 8:**
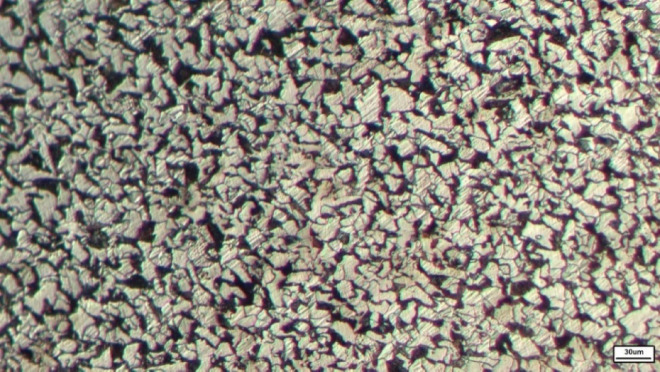
The microstructure of tie rod spindle (X200).

A sample of the tie rod spindle and head was prepared for microstructural investigation. With a carbon content of 0.2 wt %, the steel exhibits a mixed ferrite–pearlite microstructure, as shown in Fig. 8.

**Fig. 9:**
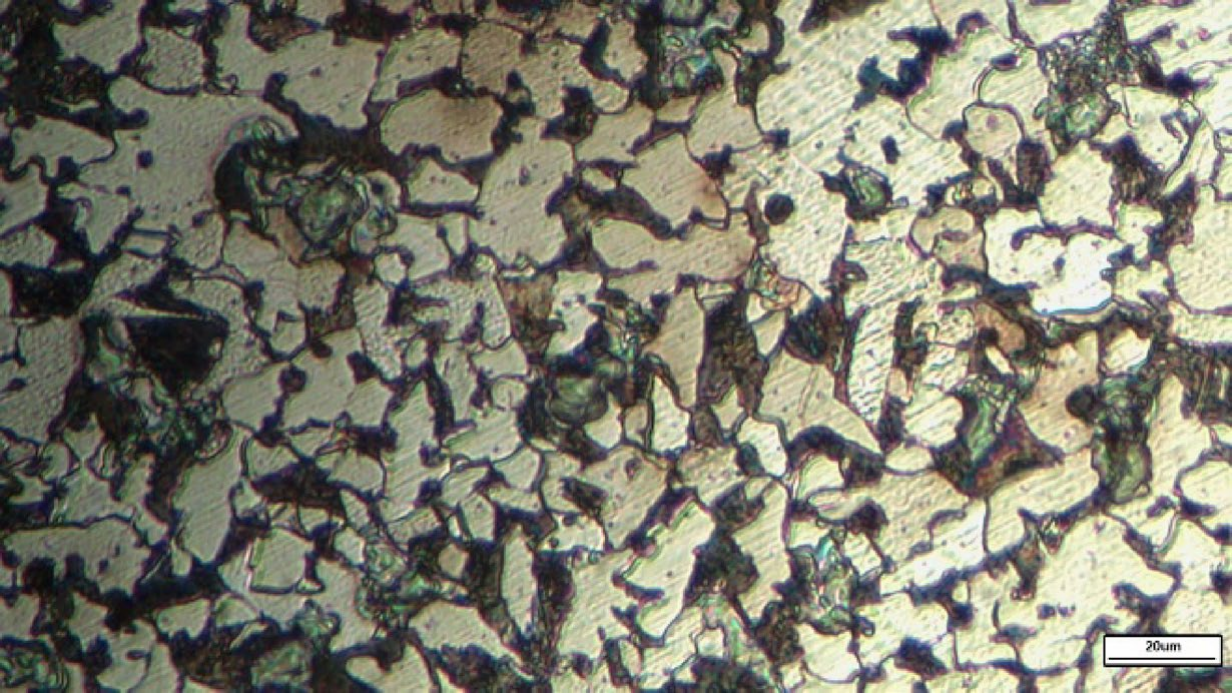
Optical microscope photo for tie rod spindle (X 500) showing bluish-green patina.

The high-magnification image (×500) in Fig. 9 confirms the expected mixed ferrite–pearlite microstructure associated with 0.2 wt% carbon content. Additionally, a scattered bright bluish-green layer is visible on the surface, likely representing a patina formed due to the presence of 0.3% copper and exposure to environmental conditions. This surface layer is distinct from the internal microstructure revealed at high magnification.

**Fig. 10:**
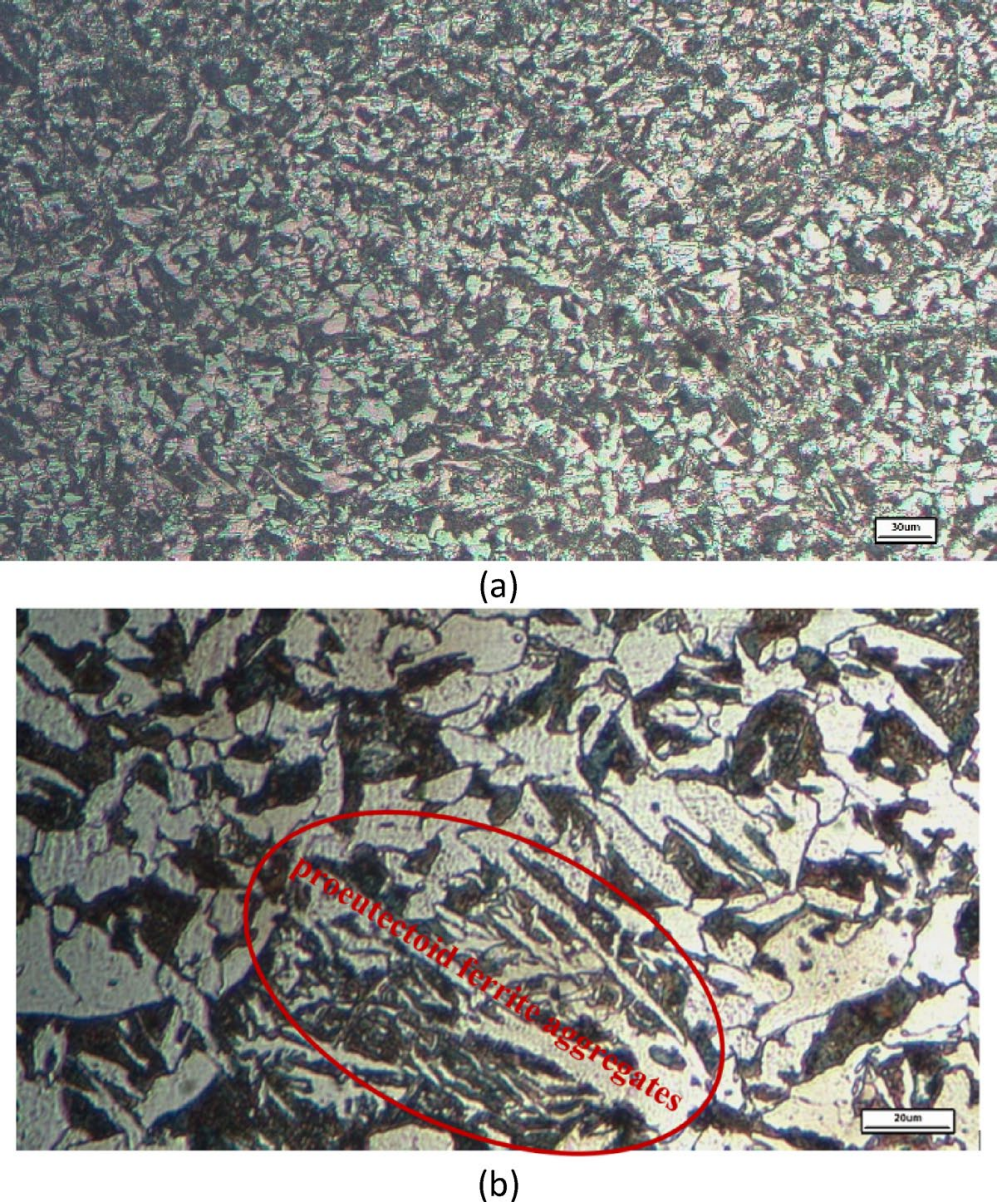
(**a**) Optical microstructure of the tie rod flange at (X 200). (**b**) Optical microstructure of the tie rod flange at high magnification (X500).

Figure 10a shows the microstructure of the tie rod flange at ×200 magnification. The material primarily consists of a ferrite–pearlite mixture, characteristic of hypoeutectoid steel with approximately 0.2 wt% carbon. The measured grain size of the specimen is 6.7 μm, corresponding to a G 15.5 rating according to ASTM E112.

Quantitative phase analysis using ImageJ indicates that ferrite constitutes 51% ± 1.3% (SD) of the microstructure, with the remainder corresponding to pearlite. The microstructure does not exhibit pronounced or continuous ferrite networks. At higher magnification (×500), shown in Fig. 10b, localized ferrite-rich regions corresponding to proeutectoid ferrite are observed^21^. The formation of these regions is attributed to heating the tie rod to 700 °C prior to deformation, followed by air cooling.

The microstructure, composed of 51% ferrite and discrete pearlite colonies with a refined, dispersed ferrite morphology, significantly enhances the mechanical properties in the flange region. This refinement increases strength while preserving good ductility^22^. The absence of continuous ferrite bands reduces directional corrosion and minimizes mechanical weak paths. As a result, the microstructure shows notable improvements in both local mechanical performance and corrosion resistance. It offers a well-balanced combination of moderate strength, high ductility, and controlled corrosion susceptibility.

Supplementary S4 illustrates an example of measuring grain size and phase distribution using ImageJ software.

### Hot pressing process and processing considerations

During hot pressing, the tie rod material undergoes significant plastic deformation to fill the die, making precise control of processing parameters essential. The initial rod cross-sectional area is 314.16 mm², and the flange volume is 18,338.6 mm³, requiring approximately 58.35 mm of rod length to be heated to achieve dimensional accuracy.

The true strain during deformation is 0.79 at a strain rate of 0.41 s⁻¹, with a deformation time of 1.9 s per piece, indicating that moderate strain rates can lead to uneven deformation.

**Fig. 11:**
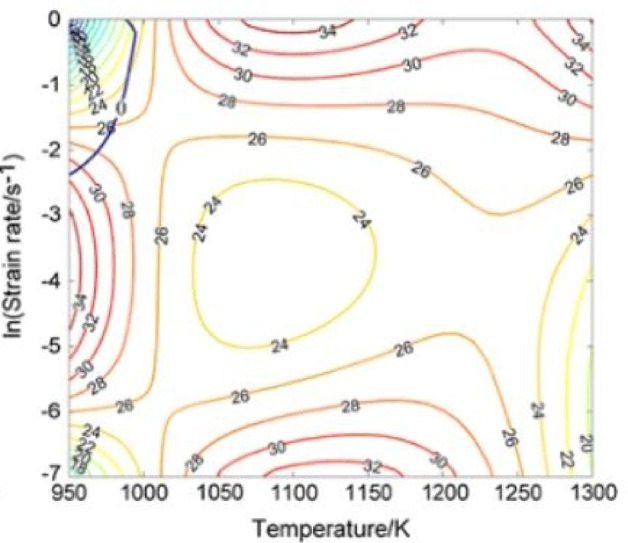
Processing maps in the isothermal compression of the 0.20% C carbon steel at strain 0.7^23^.

Processing maps are a valuable tool for designing hot-forming operations under fast or near-isothermal conditions. The processing map for plain carbon steel at a true strain of 0.7 (Fig. 11) identifies stable deformation regions between 670 °C and 1027 °C, with an optimal hot-working window at 800 °C and 0.02 s⁻¹. Maintaining deformation within this window ensures uniform flow, improved microstructure, and enhanced mechanical properties^23^.

## Conclusions

In conclusion, durable and stable tie rods are achieved by integrating optimized Cu-P alloying with precise hot deformation, enhancing corrosion resistance and uniform flange formation. The key findings are summarized as follows:

1. The tie rod is a low-alloy structural steel with copper, corresponding to EN 10,025: S355J2.
2. Copper alloying enhances corrosion resistance, with the corrosion index peaking at 0.37% Cu.
3. The steel exhibits a mixed ferrite–pearlite microstructure.
4. Stable hot-deformation regions occur between 670 °C and 1027 °C, with an optimal window at 800 °C and 0.02 s⁻¹.
5. Moderate strain rates can cause uneven flange thickness due to deformation in unstable regions.

These findings lead to durable, resource-efficient tie rods that reduce material waste and maintenance requirements. Optimized materials and processing enhance sustainable industrial practices and resilient greenhouse infrastructure.

## Supplementary Information

Below is the link to the electronic supplementary material.

Supplementary Material 1

## Data Availability

All data generated or analysed during this study are included in this published article [and its supplementary information files].
