# Supplementary material for: Optimizing weathering steel tie rod production for sustainable greenhouse structures
Source: Sci Rep. 2026 Apr 27;16:14021. doi: 10.1038/s41598-026-45791-9 (PMC13134953; doi:10.1038/s41598-026-45791-9)
Supplement: Supplementary file 1 — Supplementary Material 1 [file 41598_2026_45791_MOESM1_ESM.pdf]

Optimizing Weathering Steel Tie Rod Production for Sustainable Greenhouse Structures

Maha El-Meligy [1*], **Taher El-Bitar**[1], **Almosilhy Mohammed**[1]

Supplementary Information

Supplementary S1

Sample Preparation, Calibration, and Chemical Analysis Using Three Spark Tests

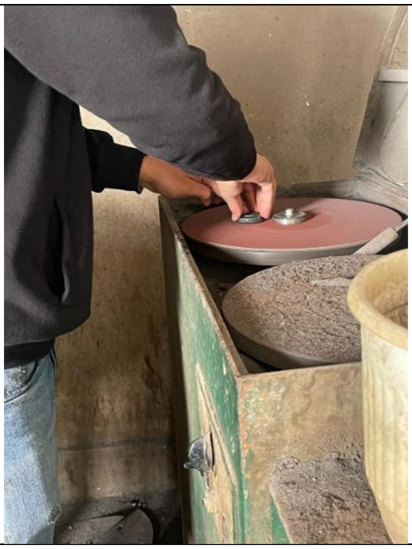	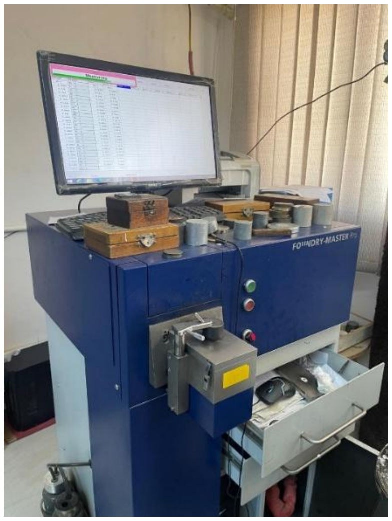
Sample preparation	Calibration process

Chemical analysis

[illegible]

Alloy : FE_100 Mode : PA 29/04/2024 02:35:18

	Fe	C	Si	Mn	P	S	[illegible]
1	98.0	0.195	0.254	0.545	0.0232	0.0226	[illegible]
2	98.1	0.184	0.250	0.574	0.0213	0.0241	[illegible]
3	98.1	0.188	0.253	0.541	0.0213	0.0231	[illegible]
**Average**	**98.1**	**0.189**	**0.252**	**0.553**	**0.0219**	**0.0233**	**0.187**

	Mo	Ni	Al	Co	Cu	Nb	[illegible]
1	0.0752	0.144	0.0192	0.0135	0.369	0.0044	[illegible]
2	0.0543	0.141	0.0174	0.0142	0.299	0.0046	[illegible]
3	0.0681	0.141	0.0187	0.0147	0.357	0.0052	[illegible]
**Average**	**0.0659**	**0.142**	**0.0184**	**0.0142**	**0.342**	**0.0047**	**0.0026**

	V	W	Pb	Sn	B	Ca	Zr
1	0.0040	< 0.0050	< 0.0040	0.0239	0.0008	> 0.0080	0.0025
2	0.0041	< 0.0050	< 0.0040	0.0234	0.0008	> 0.0080	[illegible]
3	0.0034	< 0.0050	< 0.0040	0.0233	0.0008	> 0.0080	[illegible]
**Average**	**0.0038**	**< 0.0050**	**< 0.0040**	**0.0235**	**0.0008**	**> 0.0080**	**0.0026**

	Zn	Bi	As	N	Se	Sb	Ta
1	0.0020	0.0011	0.0111	0.0096	0.0022	0.0076	[illegible]
2	0.0014	0.0015	0.0106	0.0096	0.0022	0.0010	[illegible]
3	0.0019	0.0016	0.0103	0.0096	0.0017	0.0035	[illegible]
**Average**	**0.0017**	**0.0014**	**0.0107**	**0.0096**	**0.0020**	**0.0040**	**0.0453**

Supplementary S2

Specimen Geometry and Mounting in the Universal Testing Machine (UTM) Grips

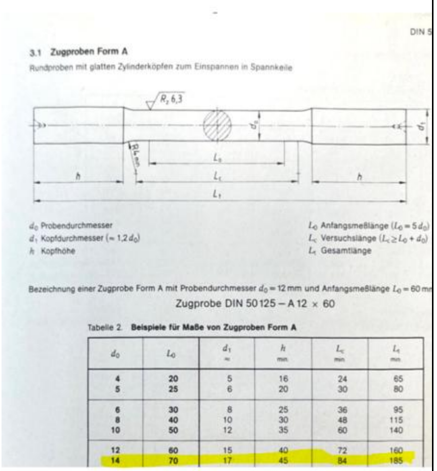

DIN 5

3.1 Zugproben Form A

Rundproben mit glatten Zylinderköpfen zum Einspannen in Spannkeile

d_0 Probendurchmesser
d_1 Kopfdurchmesser ($\approx 1,2\,d_0$)
h Kopfhöhe

L_0 Anfangsmeßlänge ($L_0 = 5\,d_0$)
L_c Versuchslänge ($L_c \geq L_0 + d_0$)
L_t Gesamtlänge

Bezeichnung einer Zugprobe Form A mit Probendurchmesser $d_0 = 12$ mm und Anfangsmeßlänge $L_0 = 60$ mm

Zugprobe DIN 50125 – A 12 × 60

Tabelle 2. **Beispiele für Maße von Zugproben Form A**

d_0	L_0	d_1 ≈	h min.	L_c min.	L_t min.
**4**	**20**	5	16	24	65
**5**	**25**	6	20	30	80
**6**	**30**	8	25	36	95
**8**	**40**	10	30	48	115
**10**	**50**	12	35	60	140
**12**	**60**	15	40	72	160
**14**	**70**	17	45	84	185

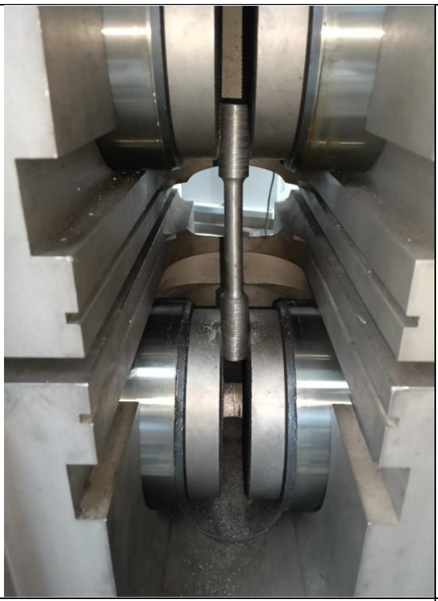

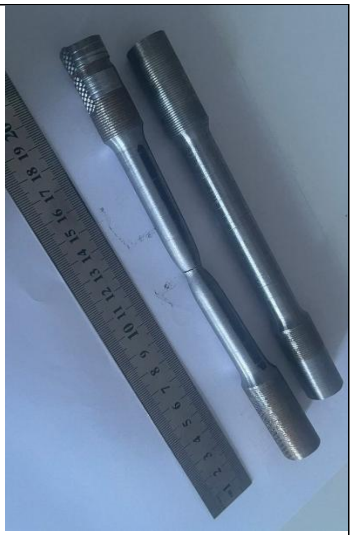

specimen geometry	specimen mounted in the grips	Specimens before &after test

Supplementary S3

Hardness Testing Machine and Test Report

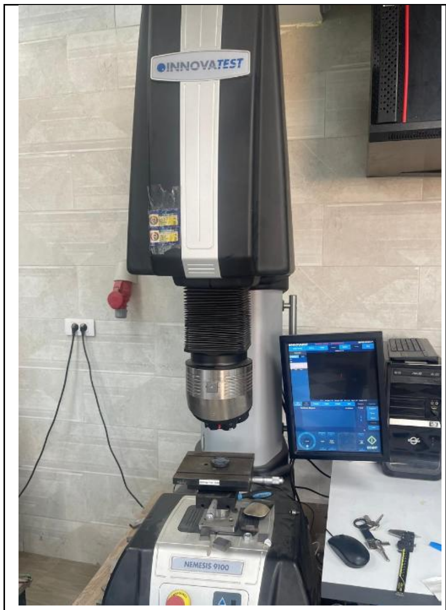

Hardness Testing Machine

Test Report

INNOVATEST Europe BV
Borgharenweg 140
6222 AA Maastricht
The Netherlands

+31 43 3520260
+31 43 3661168
info@innovatest-europe.com
www.innovatest-europe.com

General Information

**Date**	2026 ,10 فبراير
**Operator**	admin
**Hardness tester type**	NEMESIS 9104

Enter Comments

Hardness Test Information

**Method**	VICKERS
**Hardness scale**	HV10
**Dwell time**	13 sec.

Test Pattern

Diagrams

1/5

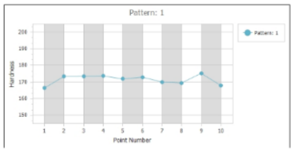

2/5

**ID**	1
**Hardness**	166.37 HV10
**d1**	0.3340 mm
**d2**	0.3337 mm
**position**	x: 0.00 mm<br>y: 0.00 mm
**Conversions**	
**Time**	11:58:23 م

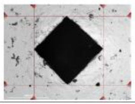

**ID**	2
**Hardness**	173.39 HV10
**d1**	0.3276 mm
**d2**	0.3264 mm
**position**	x: 0.00 mm<br>y: 0.00 mm
**Conversions**	
**Time**	12:03:19 ص

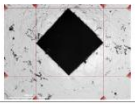

**ID**	3
**Hardness**	173.52 HV10
**d1**	0.3262 mm
**d2**	0.3277 mm
**position**	x: 0.00 mm<br>y: 0.00 mm
**Conversions**	
**Time**	12:06:03 ص

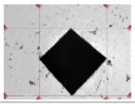

**ID**	4
**Hardness**	173.58 HV10
**d1**	0.3279 mm
**d2**	0.3258 mm
**position**	x: 0.00 mm<br>y: 0.00 mm
**Conversions**	
**Time**	12:16:11 ص

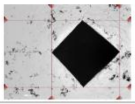

3/5

**ID**	5
**Hardness**	171.82 HV10
**d1**	0.3294 mm
**d2**	0.3277 mm
**position**	x: 0.00 mm<br>y: 0.00 mm
**Conversions**	
**Time**	12:18:36 ص

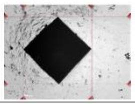

**ID**	6
**Hardness**	172.86 HV10
**d1**	0.3278 mm
**d2**	0.3273 mm
**position**	x: 0.00 mm<br>y: 0.00 mm
**Conversions**	
**Time**	12:20:54 ص

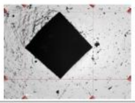

**ID**	7
**Hardness**	169.95 HV10
**d1**	0.3322 mm
**d2**	0.3284 mm
**position**	x: 0.00 mm<br>y: 0.00 mm
**Conversions**	
**Time**	12:22:31 ص

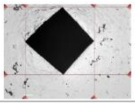

**ID**	8
**Hardness**	169.42 HV10
**d1**	0.3325 mm
**d2**	0.3291 mm
**position**	x: 0.00 mm<br>y: 0.00 mm
**Conversions**	
**Time**	12:24:17 ص

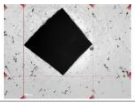

4/5

**ID**	9
**Hardness**	175.17 HV10
**d1**	0.3213 mm
**d2**	0.3294 mm
**position**	x: 0.00 mm<br>y: 0.00 mm
**Conversions**	
**Time**	12:25:51 ص

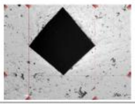

**ID**	10
**Hardness**	167.9 HV10
**d1**	0.3320 mm
**d2**	0.3326 mm
**position**	x: 0.00 mm<br>y: 0.00 mm
**Conversions**	
**Time**	12:27:28 ص

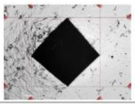

Measurement Tables

Pattern: 1

Measurement Index	Result
1	166.37 HV10
2	173.39 HV10
3	173.52 HV10
4	173.58 HV10
5	171.82 HV10
6	172.86 HV10
7	169.95 HV10
8	169.42 HV10
9	175.17 HV10
10	167.9 HV10

Statistics

Pattern	Mean	Min	Max	SD	Range	USL	LSL	Cp	Cpk
1	171.40	166.37	175.17	2.86	8.81	0.00	0.00	0.00	-20.01

5/5

Supplementary S4

Measurement of grain size and phase distribution using ImageJ software

Measurement of grain size

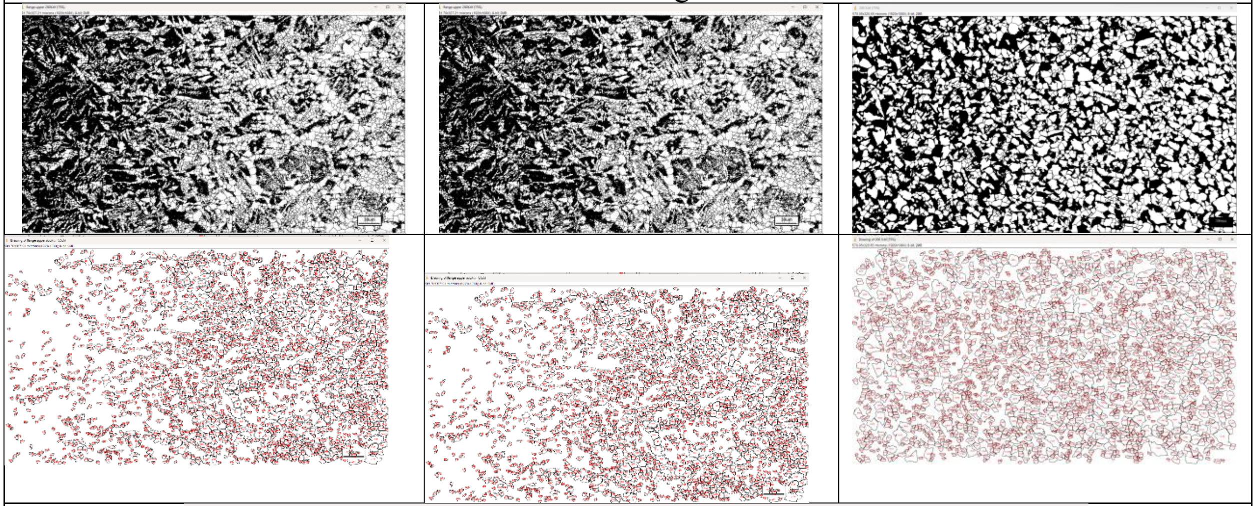

Summary.csv

File Edit Font

Slice	Count	Total Area	Average Size	%Area	Mean
flange upper 200X.tif	2323	67277.501	28.961	35.347	255
200 X-1.tif	1801	88918.800	49.372	48.608	255
upper flange 200 X-1.tif	2746	82241.633	29.950	44.139	255

$$\text{Equivalent Diameter} = 2 \times \sqrt{\frac{\text{Area}}{\pi}}$$

Mean grain size = 6.7 μm

ASTM E112 G 15.5

Analysis of ferrite %

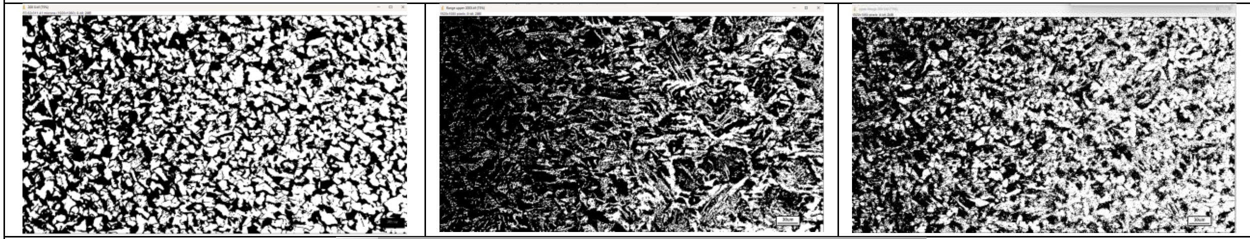

1		Min	Max	%Area
2	1	0	255	52.492
3	2	0	255	49.952
4	3	0	255	50.696
5	avarage			51.04667
6	SD			1.305804
